# Bridging the Gap: Integrated High-Density Microelectrode Arrays for Cellular, Organoid, and Clinical Electrophysiology

**DOI:** 10.3390/mi17050611

**Published:** 2026-05-15

**Authors:** Qinghua Wu, Yan Gong, Xiang Liu

**Affiliations:** 1 Department of Anesthesiology, The First Hospital of Putian City, Putian 351100, China; wqhpt07@outlook.com; 2 The Biomedical Microsystems Laboratory, Alfred E. Mann Department of Biomedical Engineering, University of Southern California, Los Angeles, CA 90089, USA; yangong@usc.edu; 3 Department of Radiology, Michigan State University, East Lansing, MI 48824, USA

**Keywords:** microelectrodes array, high-density, electrophysiological signal, electrical recording, neural interface, closed-loop, brain-machine interface, organoids, neural modulation

## Abstract

High-density microelectrode arrays (HDMEAs) have become increasingly important tools in neuroscience and biomedical engineering because of their high spatial and temporal resolution for recording and modulating electrical activity across diverse biological systems. Initially developed for in vitro studies of cultured cells, HDMEAs are now being applied to increasingly complex models, including organoids, animal systems, and even human neural systems. These advancements enable a deeper investigation of cellular interactions, network dynamics, and disease mechanisms, as well as providing novel therapeutic and diagnostic tools for neurological disorders. This review explores the evolution of HDMEAs, emphasizing recent innovations in their design, fabrication, and functionalization. We discuss their applications across cellular models, organoid systems, animal studies, and human electrophysiology, and highlight current challenges such as biocompatibility, long-term stability, scalability, and translational deployment. Finally, we outline future directions for advancing HDMEA technologies in both research and clinical settings.

## 1. Introduction

High-density microelectrode arrays (HDMEAs) have revolutionized the interface between advanced technology and biological systems, extending their influence beyond fundamental research into the realms of human health and clinical medicine [1,2,3,4,5,6,7,8,9]. These devices offer unparalleled precision in recording and manipulating the electrical activity of cellular and neural networks, enhancing our understanding and treatment of complex biological and medical challenges. HDMEAs’ ability to perform detailed electrophysiological monitoring at both the cellular and subcellular levels has ushered in new methodologies for exploring the intrinsic properties of the nervous system, cardiac tissue, and muscular functions [1]. This technology supports a broad spectrum of applications, from studying cellular interactions and network behaviors in laboratory settings to deploying advanced neural interfaces that improve clinical outcomes in disease diagnosis, prosthetic control, and human–machine communication [10,11,12,13].

The significance of HDMEAs lies not only in their recording density but also in their adaptability across biological scales. In cellular models, HDMEAs allow for real-time monitoring of electrophysiological properties, providing critical insights into cellular behavior [14], drug screening [15], and disease modeling [16]. Organoid applications leverage HDMEAs to study three-dimensional tissue constructs, replicating physiological environments for brain, cardiac, and other organ systems [17]. This approach enables researchers to track developmental processes and pathological changes over extended periods [18]. In animal models, HDMEAs facilitate long-term recordings from neural and muscular tissues, supporting applications such as electrocorticography (ECoG) [19,20,21], electromyography (EMG) [22,23,24], and cardiac electrophysiology [25,26,27], providing critical insights into network dynamics and disease mechanisms in vivo. In human applications, HDMEAs are increasingly employed for clinical diagnostics and therapeutic interventions, offering high-resolution recordings in brain-machine interfaces (BMIs), intraoperative neuromonitoring, and cardiac electrophysiology [1]. A particularly groundbreaking application of HDMEAs is in the development of closed-loop brain-machine interfaces (BMIs) [1], which enables real-time interaction between neural signals and external devices to dynamically respond to physiological and/or pathological changes and provide novel therapeutic solutions for severe neurological conditions [28].

**Figure 1:**
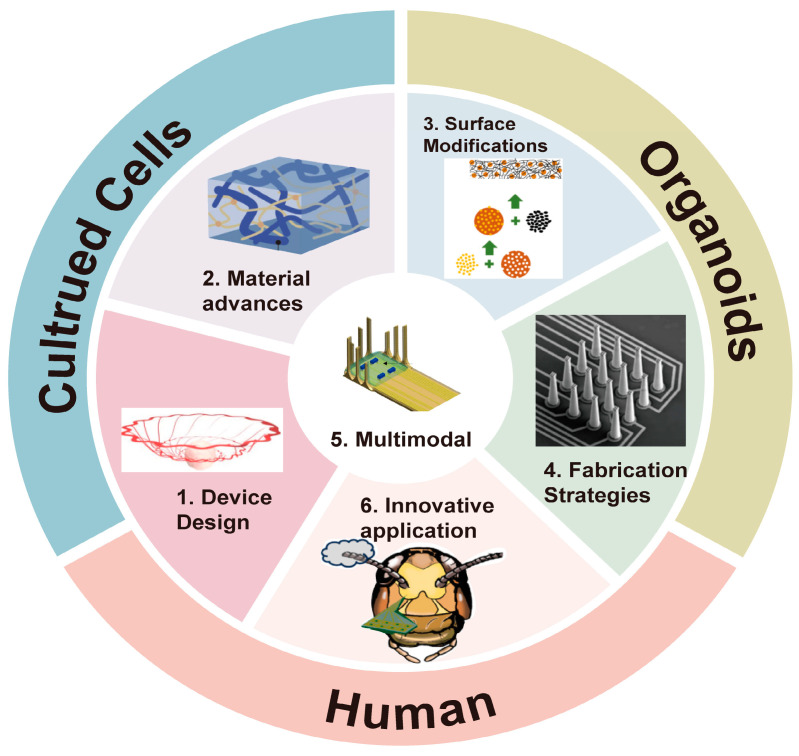
Advancements and Applications of HDMEAs Across Biological Systems. The schematic summarizes recent progress in HDMEAs across materials, device design, surface modification, fabrication, innovative applications, and multimodal integration. (1) Device design: Kirigami electronics enable deformable interfaces for long-term recording from human neural organoids and assembloids [29]. (2) Material advances: PEDOT:PSS/PVA double-network hydrogels illustrate soft, stretchable, and conductive materials for compliant bioelectronics [30]. (3) Surface modifications: Drug-loaded silica nanoparticles embedded in PEDOT films demonstrate multifunctional electrode coatings for recording and neurochemical modulation [31]. (4) Fabrication strategies: Direct laser writing enables 3D microelectrodes and conductive traces on flexible substrates [32]. (5) Multimodal: A folded 3D opto-electro array integrates optical stimulation with electrophysiological recording [33]. (6) Innovative application: A flexible dual-sided MEA records locust olfactory neural responses for VOC-based human lung cancer detection [34].

In recent years, advances in materials, device architectures, and fabrication strategies have improved the flexibility, biocompatibility, and scalability of HDMEAs, facilitating their use in applications that extend beyond conventional in vitro experiments [10,11,12,13] (as shown in Figure 1). HDMEAs are increasingly being integrated into multimodal and closed-loop systems, supporting applications such as neural interfacing, intraoperative monitoring, prosthetic control, and brain–machine interfaces [1,28]. These developments highlight a broader transition in the field: from isolated recording arrays toward integrated bioelectronic platforms capable of bridging the gap between controllable experimental models and clinically relevant human electrophysiology.

In this review, we examined HDMEAs from a cross-scale perspective, with an emphasis on how device requirements, interface strategies, and functional capabilities evolve from cellular models to organoids, animal models, and human applications. It focuses on how HDMEA design requirements, interface strategies, and functional roles change across biological scales, from in vitro systems to clinically relevant human applications. We first discuss how HDMEAs can be defined across applications and how their signal modalities and integrated functionalities have expanded over time. We then summarize the key design requirements and technological drivers that shape HDMEA development across biological scales, followed by a review of their applications in in vitro cellular systems, organoids and engineered tissues, in vivo models, and clinical electrophysiology. Finally, we highlight the major bottlenecks and future opportunities for developing standardized, multimodal, and clinically deployable HDMEA platforms.

## 2. HDMEAs as Cross-Scale Bioelectronic Interfaces

MEAs and HDMEAs were initially developed to detect extracellular electrophysiological signals primarily by detecting electrical activity generated by excitable cells such as neurons or cardiomyocytes [35,36]. However, recent studies have expanded this view by showing that MEAs can also capture electrode signal changes unrelated to active cellular discharge, including dynamic real-time impedance variations associated with cell adhesion, contraction, viability, and other interface-dependent behaviors [37,38]. In Table 1, we summarize representative signals that could be detected by MEAs along with their key characteristics.

### 2.1. Definition and Variability of HDMEAs

The term HDMEAs encompasses descriptors such as high spatial resolution, multi-channel, and high temporal resolution [47,48]. Spatial resolution relates to the number of neurons that can be engaged (channel count or density), while temporal resolution reflects sampling rate and bandwidth. Despite the widespread use of the term, a standardized definition for HDMEAs has long been absent. As a result, devices with markedly different geometries and channel densities have all been described as HDMEAs. Existing studies label MEAs as high density, with electrode densities ranging from less than one channel/mm^2^ to several thousand channels/mm^2^ [1]. This variability arises because different biological systems impose different recording demands.

Neural recordings often demand higher densities than muscular recordings. For neuronal recordings, neurons in the murine cortex have an average density of ~105 neurons/mm^3^, corresponding to a theoretical HDMEA density of ~2.5 × 10^3^ channels/mm^2^ (pitch 100 μm) to match neuron spacing of ~20 μm [6,49,50]. Thus, ideal HDMEAs for neural recording entail electrode site spacing of ≤100 μm.

Conversely, motor unit recordings target areas spanning several thousand square microns [22]. For muscular recordings(electromyography), HDMEAs were previously defined by electrode spacing of a few millimeters [51].

Technological differences also contribute to density variations. Silicon-based probes can achieve thousands of channels per mm^2^ [52], while flexible HDMEAs typically exhibit lower densities, constrained to tens to hundreds of channels per mm^2^ [1]. Likewise, design choices—such as stretchable configurations—may reduce channel density compared to conventional flexible planar MEAs. Additionally, three-dimensional, multilayer, or dual-sided designs may prioritize volumetric coverage or conformability rather than simply maximizing planar electrode density.

As summarized in Table 2, state-of-the-art HDMEA platforms span a broad design space, with channel counts ranging from single-digit arrays to more than 26,000 electrodes, electrode sizes from a few micrometers to hundreds of micrometers, and pitch values from sub-10 μm spacing to several hundred micrometers, depending on the target application and interface geometry. These devices have been developed not only for neural electrophysiology, such as cortical mapping, brain–computer interfaces, and epilepsy-related recordings, but also for muscular recording, cardiac electrophysiology, arrhythmia analysis, retinal prostheses, neurochemical sensing, intraoperative monitoring, and multimodal bioelectronic assays. Together, these examples highlight that HDMEA design is strongly application-dependent, with channel density, electrode dimensions, and spacing optimized according to the biological scale, signal modality, and functional demands of each use case. In this review, the term HDMEA therefore encompasses not only conventionally defined high-density neural recording arrays, but also representative high-resolution and multifunctional microelectrode platforms that provide sufficiently dense spatial sampling for their intended biological and bioelectronic applications.

### 2.2. Signal Modalities and Interface Mechanisms

#### 2.2.1. General Principles for Electrophysiological Recording by MEAs

The recording process is fundamentally governed by signal generation, propagation through the conductive extracellular environment, and transduction at the electrode interface [36]. Extracellular ionic currents give rise to local potential changes that are coupled to the electrode surface, where the electrical double layer and interface impedance determine how effectively these signals are converted into measurable voltage changes with resolution.

The recording process involves four primary steps [36]: (1) Signal Generation—APs from excitable cells generate extracellular electric fields. (2) Propagation—These signals spread through the conductive medium to reach electrodes. (3) Electrode Polarization—Ionic currents at the electrode–electrolyte interface are converted into measurable voltage changes, allowing the electrode to capture extracellular signals such as action potentials and local field potentials. (4) Amplification and Processing—Weak signals (several–500 µV) are amplified, filtered, and analyzed to extract waveform, frequency, and peak characteristics.

#### 2.2.2. Equivalent Circuit Considerations at the Cell–Electrode Interface

A simplified equivalent-circuit framework is often used to interpret MEA recordings at the cell–electrode interface [36]. In this view, the interface can be described by the resistance of the surrounding extracellular medium, the seal resistance between the cell membrane and the electrode surface, and the impedance of the electrode–electrolyte interface, which is commonly represented by a combination of double-layer capacitance and charge-transfer resistance [36]. Under these assumptions, extracellular signals are not measured as direct intracellular membrane potentials, but as local potential changes transmitted through the conductive extracellular space and filtered by the interface impedance. Accordingly, signal amplitude, bandwidth, and signal-to-noise ratio depend not only on the biological source of activity, but also on electrode geometry, cell–electrode distance, and the quality of coupling at the interface [48].

#### 2.2.3. Impedance-Based Monitoring in MEAs

Electrode signal changes unrelated to active cellular electrical discharge refer to measurements that capture passive electrical variations at the cell-electrode interface, such as impedance changes reflecting adhesion, viability, or mechanical activity. Recently, Chen et al. [37] demonstrated a novel flexible electrode integrated into a Transwell system capable of real-time impedance monitoring of cardiomyocytes. This system enabled non-invasive assessment of cell adhesion, viability, and electrophysiological activity, while also providing electrical stimulation to study cardiomyocyte behavior. Similarly, Schmidt et al. [38] developed a high-density microelectrode array (HD-MEA) with integrated impedance spectroscopy to monitor mechanical contractions of human iPSC-derived cardiomyocytes. Their system achieved multimodal monitoring of cardiac function, combining field potential mapping and mechanical impedance measurements, and provided new insights into arrhythmogenic rotor patterns through label-free methods. These studies highlight the growing utility of non-discharge-based electrode signals in evaluating cellular behavior and mechanical-electrical coupling.

#### 2.2.4. Integration of Recording MEAs into Multimodal Systems

Recent advances have increasingly transformed MEAs from recording-only devices into multimodal platforms capable of simultaneous sensing, stimulation, and functional interrogation. In addition to extracellular electrophysiological recording, integrated MEA systems may incorporate optical stimulation and imaging, neurochemical sensing, impedance spectroscopy, and electrical stimulation to capture biological activity more comprehensively [17,18,33]. Representative examples include high-density transparent graphene arrays that enable combined electrical recording and optical readout for neural activity mapping [61], dual-mode microchips that support simultaneous electrophysiological and neurochemical measurements [74], and multimodal cardiac HDMEA platforms that integrate field-potential mapping with impedance-based mechanical sensing for arrhythmia analysis [38]. Representative state-of-the-art HDMEAs and integrated MEA-based microsystems are summarized in Table 2, with emphasis on electrode materials, sensing and stimulation modalities, and major application scenarios.

## 3. Ideal Properties, Advances, and Gaps in HDMEA Development

This section builds upon our previous work that focused on flexible HDMEAs for closed-loop brain-machine interfaces (BMIs) [1,75], where we explored the critical role of flexibility in enhancing the compatibility of HDMEAs with neural tissues and the unique advantages of closed-loop systems in delivering adaptive and targeted therapeutic stimulation. Here, we broaden the scope to discuss both flexible and rigid HDMEAs, addressing their application in open and closed-loop systems, with a focus on the ideal properties, challenges, and development strategies relevant to HDMEA technology.

### 3.1. Ideal Properties and Application-Dependent Requirements

Although the requirements for HDMEAs may vary depending on the application, several key general properties are critical: Firstly, high spatial and temporal resolution is essential for resolving electrophysiological activity with sufficient precision, particularly for monitoring single-neuron activity, mapping local circuits, and characterizing neuronal population dynamics [1,76]. Secondly, mechanical compliance, achieved through flexible substrates such as parylene or polyimide, minimizes the mechanical mismatch between the array and soft neural tissue [1,77,78], significantly reducing inflammation and mechanical damage risk to ensure stable long-term recordings [13,29,78,79,80,81,82]. Thirdly, biocompatibility is essential to minimize immune responses and scar tissue formation that can degrade signal quality over time. Using biocompatible materials like PEDOT:PSS, graphene, and polyimides enhances interface stability and ensures long-term functionality, which is particularly vital for chronic applications [30,83,84]. Furthermore, a high Signal-to-Noise Ratio (SNR) is critical for detecting neural signals amidst background noise, enabling HDMEAs to capture even the faintest activities, with noise reduction further achieved through advanced materials and electrode designs [85,86,87,88,89]. For chronic implants, long-term stability is vital, with coatings such as iridium oxide and carbon nanotubes enhancing electrode longevity by improving charge injection capacity and minimizing corrosion. Modern HDMEAs are increasingly multimodal, capable of integrating electrophysiological, optical, and biochemical sensing for comprehensive monitoring of neural activity and neurochemical dynamics. Finally, scalable fabrication techniques, including photolithography and 3D printing, enable the production of HDMEAs at reduced costs, which is crucial for transitioning the technology from research to widespread clinical use [77,90].

Other than the general properties, there are model-specific expected properties for HDMEAs in cellular models, organoids, animal models and human applications.

#### 3.1.1. Cellular Models

In cellular models, the ideal properties of HDMEAs center around to achieve higher spatial resolution and electrode density than in vivo models, so that to capture single-cell and subcellular electrophysiological activity. This fine resolution is critical for monitoring individual cells, mapping network dynamics, and conducting high-throughput drug screening [2]. Non-interference recording capabilities are essential to preserve cell viability, allowing repeated measurements without disrupting cellular functions [91]. HDMEAs for cellular applications must also exhibit excellent chemical stability, maintaining performance and signal fidelity even during long-term exposure to biological culture environments. Additionally, scalability is a key factor, enabling large-scale recordings from multiple cells while maintaining precision and minimizing cross-talk between electrodes. Integration with microfluidic systems and other modules further enhances the versatility of HDMEAs, allowing for dynamic control of the cellular environment and facilitating lab-on-chip platforms for advanced cellular assays [92,93].

#### 3.1.2. Organoids

HDMEAs for organoid models require 3D conformability to effectively interface with the curved and irregular surfaces of organoids, ensuring comprehensive spatial coverage for accurate signal capture [76,94]. This adaptability allows for seamless integration with the organoid’s structure, supporting surface and deep-layer recordings. Layer-specific recording capabilities are essential for monitoring activity at different depths within the organoid, providing valuable insights into internal network formation and tissue maturation [95,96]. To fully harness the potential of organoids, real-time monitoring of biological states and functional evaluation of organoids are crucial [17,18]. Chronic monitoring is a key property, enabling long-term observation without disrupting organoid growth or function, which is critical for studying developmental processes and disease progression over extended periods [29,76]. Multimodal and functional integration further enhances HDMEAs’ utility, allowing simultaneous electrophysiological, optical, and biochemical measurements to gain a more complete understanding of organoid behavior [1]. Additionally, reusability is a desirable feature, ensuring that HDMEAs can be applied across multiple experiments, reducing costs and enhancing the efficiency of long-term organoid studies [1].

#### 3.1.3. Animal Models

In vivo applications place stronger emphasis on implantation feasibility, mechanical durability, chronic stability, and reliable tissue integration. For neural and muscular recordings in animal models, HDMEAs must maintain high-quality signal acquisition despite motion, micromotion, and foreign-body responses, while minimizing tissue disruption during insertion and long-term use [97,98,99]. Thus, flexible substrates, bioactive coatings, and insertion-assist strategies become increasingly important design considerations at this stage.

#### 3.1.4. Human and Clinical Applications

Ideal HDMEAs for human applications must prioritize biocompatibility and long-term safety to ensure stable in vivo performance without triggering immune responses or adverse tissue reactions. Single-unit resolution and high-quality recording is crucial for capturing intricate cellular signals, supporting advanced diagnostics, and precise therapeutic interventions [100]. Mechanical flexibility and durability are essential, allowing the electrodes to conform to soft tissues and minimizing the risk of micromotion-induced damage or signal disruption in dynamic environments such as the brain or heart. Wireless and minimally invasive designs improve patient comfort and mobility, enabling real-time monitoring and data transmission without the need for external connectors or bulky equipment [101]. Closed-loop feedback systems play a vital role by providing adaptive therapeutic interventions, dynamically adjusting stimulation or recording parameters in response to physiological changes—critical for applications [1,17,102]. Additionally, HDMEAs must seamlessly integrate with existing clinical diagnostic tools, including X-ray, CT, PET, and MRI, ensuring compatibility with current medical workflows and enhancing their practicality for widespread clinical adoption.

### 3.2. Key Considerations, Advance and Gaps in Developing Ideal HDMEA

The development of HDMEAs is constrained by intertwined mechanical, electrical, chemical, and biological factors, all of which shape device performance, signal stability, and translational potential [1,47,103,104]. The realization of ideal HDMEAs necessitates careful consideration of materials, fabrication strategies, design geometries, and surface modifications, all of which influence signal quality, stability, and the ease of implantation. As summarized in Table 2, recent HDMEA platforms vary substantially in electrode materials, device architectures, sensing modalities, and stimulation functions, underscoring that HDMEA design is closely tied to application-specific performance requirements.

**Material Considerations:** The choice of materials for HDMEAs is paramount in determining biocompatibility, electrical performance, and mechanical compliance. Materials must be conductive, durable, and flexible to minimize the mechanical mismatch between the electrode and soft neural tissues [1,6,105]. Traditional materials like platinum and iridium exhibit excellent conductivity and biocompatibility but often lack flexibility. Advancements have shifted towards polymers (polyimide, Parylene-C) and conductive coatings like PEDOT:PSS, which reduce inflammatory responses while preserving high signal fidelity [106,107,108,109] and carbon nanotubes [110,111,112] offer promising alternatives due to their exceptional mechanical strength and low impedance, contributing to improved signal quality. Diamond materials, known for their mechanical robustness, biocompatibility, and chemical inertness, are ideal for long-term in vivo bioelectronics and HDMEAs, offering high thermal conductivity, low electrical noise, and surface functionalization capabilities that enhance cellular adhesion, reduce biofouling, and ensure stable, high-quality electrophysiological recordings [15,113].

**Fabrication Strategies:** Fabrication techniques play a vital role in achieving the high spatial resolution necessary for HDMEAs. Traditional photolithography and etching techniques allow for the creation of precise microelectrode configurations; however, they are confined to the microscale, and their complexity and cost pose significant challenges [15,54,114,115,116]. Among current techniques to craft flexible HDMEAs, the double-sided configuration is a promising solution, not only doubling the channel count without increasing the size of the probe but also enabling signal recording from both sides [1,67]. Kim et al. [67] and Pimenta et al. [117] combined multi-layer designs with sacrificial metal layers to fabricate dual-sided probes. While this approach offers the potential for complex electrode configurations, challenges remain in achieving robust attachment between the sacrificial metal layer and the carrier wafer, as well as ensuring strong adhesion between the electrode metal layer and the isolation layer. Liu et al. [34,77,90] introduced a cost-efficient fold-annealing method that enables the creation of flexible, high-density, dual-sided microelectrode arrays (MEAs), which facilitate neural recording from both sides. This technique achieves unprecedented electrode densities of 463, 687, and 766 channels/mm^2^, with center-to-center spacings of 50–70 μm on one side and an average of 25–35 μm across both sides of a single probe. While cleanroom-based microfabrication remains the gold standard, recent efforts have focused on scalability. For instance, Kim et al. [118] demonstrated the use of industrial flexible PCB technology to fabricate 3D-protruded electrodes, offering a cost-effective pathway for mass production.

The integration of complementary metal-oxide-semiconductor (CMOS) processes has facilitated the production of ultrahigh-density electrode arrays, though these require complex and expensive infrastructure [97,115,116,119,120,121,122]. Emerging techniques, such as 3D printing and additive manufacturing, present opportunities for customizable, cost-effective HDMEAs, fostering greater accessibility and innovation [1,32,105].

Additionally, most HDMEAs are still manually assembled, requiring significant labor and leading to inconsistencies in device performance. Automating this process through additive manufacturing and robotic systems could streamline production and improve reproducibility [123,124,125].

**Design Geometries and Surface Modifications:** The structural design and geometry of HDMEAs directly impact their recording capabilities and implantation success [105,126,127]. Flexible designs conform to brain surfaces, reducing micromotion-induced damage, but these designs are often susceptible to buckling during implantation [97,98,99].

At the same time, smaller electrodes improve spatial selectivity but generally increase impedance, making surface modification critical for preserving signal quality. Strategies such as platinum black, iridium oxide, PEDOT-based coatings, nanostructured gold, and bioactive surface treatments have therefore been widely explored to improve coupling efficiency, reduce noise, and modulate tissue response [128,129,130]. Even so, maintaining stable performance over time remains challenging because of biofouling, corrosion, signal drift, and chronic foreign-body reactions [82,131,132].

**Facilitating the Implantation Process:** The implantation process for MEAs must balance mechanical rigidity with flexibility to prevent damage to neural tissues [133,134]. Flexible electrodes are advantageous for chronic implants but present challenges during insertion [48,134]. Solutions such as the use of stiffening shuttles, bioresorbable coatings, and magnetic actuation techniques have emerged to enhance implantation precision and minimize trauma [99]. Additionally, robotic implantation systems are being developed to automate insertion, ensuring accuracy and reducing the risk of human error [135].

Taken together, the advancement of HDMEAs requires a multidisciplinary approach, integrating materials science, microfabrication, and neuroengineering. Addressing key challenges in material selection, fabrication techniques, and implantation processes will pave the way for next-generation HDMEAs with enhanced signal quality, long-term stability, and broad clinical applicability.

## 4. Advances and Applications of HDMEAs in Cultured Cell Models

In cell culture systems, HDMEAs support not only high-resolution electrophysiological recording [136], but also a broader range of bioelectronic assays, including drug response profiling, electromechanical coupling analysis, neurochemical sensing, and interface-dependent cellular measurements. These relatively controlled in vitro settings have therefore become important platforms for both device validation and high-content functional analysis.

### 4.1. Structural and Design Innovations in HDMEAs for Cells

Recent advances in cellular HDMEA design have focused on improving compatibility with in vitro culture platforms while expanding the ability to record, stimulate, and manipulate cellular activity. For example, Chen et al. [37] introduced a flexible electrode integrated with a transwell system for in situ real-time monitoring and regulation of cardiomyocyte electrophysiology. This design allows for seamless integration with existing cell culture platforms, enabling continuous tracking of cellular responses to electrical stimulation. Sato et al. [137] further combined HDMEAs with a modular microfluidic platform to construct defined neuronal networks, highlighting the value of integrating microfluidic engineering with large-scale electrophysiological recording.

At the same time, cellular HDMEAs have increasingly incorporated multimodal and perturbative functions. Kobayashi et al. [136] combined HDMEAs with optogenetics to reveal interactions between single-neuron and network-wide activity, providing insight into neuronal networks and epileptic seizure mechanisms. This study exemplifies how HDMEAs can simultaneously stimulate and record neural activity at a single-neuron level. Miyahara et al. [138] developed a hypersensitivity evaluation method using HDMEAs to record neuronal activity alterations in cultured sensory neurons, contributing to pain research. Parodi et al. [93] used an commercial MEAs with 2304 electrodes (for each well characterized by 60 µm in pitch and 25 µm in electrode size) to detect the extracellular signal traces. Together, these studies show that structural innovation in cellular HDMEAs is increasingly directed not only toward higher-density recording, but also toward better integration with stimulation, microfluidics, and multimodal assay platforms.

### 4.2. Enhancing Cellular Analysis

HDMEAs have expanded cellular electrophysiology beyond conventional extracellular voltage recording by enabling multimodal and higher-content functional analysis. In cardiomyocyte studies, Schmidt et al. [38] advanced this by creating a 512-electrode multilayer HDMEA capable of monitoring re-entry arrhythmias and mechanical contractions in hiPSC-derived cardiomyocytes. This array demonstrated sensitivity to cardioactive drugs and successfully detected rotor patterns indicative of arrhythmias.

Beyond excitable cells, HDMEAs have also been used to detect non-classical cellular signatures. Ell et al. [46] demonstrated the use of HDMEAs for label-free identification of non-electrogenic cancer cells by measuring adhesion noise, providing novel insights into cancer cell detection and analysis. These innovations in electrode design facilitate improved sensitivity and recording fidelity for cellular studies. In parallel, Mulberry et al. [74] developed a dual-mode microelectrode array capable of simultaneous electrophysiological and neurochemical measurements, bridging the gap between electrical and chemical analyses of cultured cells. This system offers comprehensive insights into neurotransmission and synaptic dynamics, crucial for studying neuronal networks and cardiomyocytes.

These expansion is important because it moves cellular HDMEA applications beyond a predominantly neural framework toward broader bioelectronic assays relevant to toxicity screening, non-electrogenic cell characterization, and multimodal phenotyping.

### 4.3. Applications in Neural Network Analysis, Disease Models, and Drug Screening

HDMEAs have become important tools for studying both single-cell and network-wide electrophysiological activities in cultured cells. Kobayashi et al. [136] employed HDMEAs combined with optogenetic stimulation to investigate single-neuron and network-wide interactions in cultured cortical neurons. This experimental setup enabled simultaneous single-neuron and network-level recordings, identifying mechanisms underlying epileptic seizures and highlighting the role of leader neurons in initiating network bursts. Ramezani et al. [61] introduced transparent graphene HDMEAs capable of predicting cellular calcium activity, correlating surface potential recordings with calcium imaging for neural network studies. This multimodal approach advances disease modeling and neurodegenerative research by enabling simultaneous electrical and optical recordings.

HDMEAs have been employed to study neuronal networks and synaptic transmission at high spatial and temporal resolutions. Yoon and Nam [95] developed a 3D neuronal network read-out interface that utilizes neuronal cluster patterning on microelectrode arrays, achieving high-performance recordings from cultured neural networks. This platform facilitates the investigation of neural connectivity, network plasticity, and pathophysiological conditions, offering new possibilities for neurodegenerative disease research. Emery et al. [115] expanded the application of HDMEAs by demonstrating large-scale biosensors capable of recording synaptic transmission and long-term potentiation (LTP) in hippocampal networks. These systems enable detailed analysis of hippocampal circuits, providing a valuable tool for studying memory formation and neural plasticity in vitro. Similarly, Sato et al. [137] engineered modular neuronal networks on HDMEAs, enhancing the functional complexity of neural circuits through microfluidic cell engineering.

Applications have also expanded into disease modeling and drug screening, particularly in human induced pluripotent stem cells (iPSC)-derived cardiomyocyte systems. For example, Zhang et al. [92] utilized HDMEAs to study the electrical and mechanical coupling of hiPSC-CM layers, unveiling key differences in cardiomyopathies such as Brugada syndrome and dilated cardiomyopathy. By integrating atomic force microscopy (AFM) and traction force microscopy (cTFM), this study achieved high-throughput mechanical measurements alongside electrophysiological mapping [92]. Spanu et al. [139] introduced a micro organic charge modulated array (MOA) to simultaneously record electrical and metabolic activity in cardiac cells, presenting a comprehensive solution for monitoring cellular responses to drug treatments. Lee et al. [140] introduced CardioMEA, a comprehensive data analysis platform tailored for HD-MEA data from hiPSC-CMs. CardioMEA facilitates scalable data processing, visualization, and analysis, addressing the need for efficient electrophysiological and pharmacological data interpretation. The platform was successfully applied to assess the effects of antiarrhythmic drugs on hiPSC-CM cultures, demonstrating its potential in cardiac disease modeling and drug screening.

## 5. Advances and Applications of HDMEAs in Organoid Research

The development of in vitro 3D cell culture technology has led to the emergence of 3D biological constructs (e.g., organoids, spheroids, and assembloids) that are closer to the physiological properties of human organs. As a self-organized three-dimensional structure, organoids are derived from pluripotent stem cells or isolated organ progenitors, which contain at least one cell type in the target organ and can self-assemble into organ-like structures with their physiological structure and functional characteristics [141]. HDMEAs have become pivotal in the realm of organoid research, offering a transformative approach to studying these 3D cellular structures, including brain, cardiac, retinal, and taste organoids [1,96,142,143,144,145].

### 5.1. Traditional Two-Dimensional (2D) MEAs for Organoid Research

Traditional 2D MEAs are widely used to detect and characterize electrophysiological properties of organoids by placing organoids directly on top of the microelectrode array. This method enables the detection of extracellular action potentials, local field potentials, and network signals from neurons at the organoid base [144].

2D-HDMEAs embedded in wells capture data in two dimensions, allowing researchers to infer complex three-dimensional interactions within various organoid cultures through advanced data analysis. Trujillo et al. [146] recorded both single- channel and population firing characteristics derived from the local field potential (LFP)through an 8-well MEA plate, in which each well contained 64 low-impedance (0.04 MΩ) platinum microelectrodes. The electrodes had a diameter of 30 µm and were spaced 200 µm apart. The recordings captured the spontaneous electrical activity of the organoids weekly, starting two weeks post-plating, over a 10-month period, demonstrating that brain organoids generated more stable and robust electrical activity over 40 weeks compared to iPSC-derived planar neuron cultures, highlighting the advantages of organoid-based models for long-term studies. Yoon and Nam [95] introduces a neuronal cluster interface to improve the coupling between microelectrodes in 2D MEAs and 3D neuronal networks. By patterning small neuronal clusters directly onto microelectrodes, the interface facilitates stable, long-term electrophysiological recordings from 3D networks, enhancing the active channel ratio and signal-to-noise ratio (SNR) for up to a month. This method offers a cost-effective alternative to complex 3D MEAs, enabling simultaneous recording and stimulation of 3D neural structures without altering their activity characteristics, thus expanding applications in studying neural organoids and 3D neuronal models. Deng et al. [147] explored the application of electrical impedance imaging using HD-MEAs for non-invasive monitoring of cell cultures. They developed practical electrical impedance tomography (EIT) strategies for 3D imaging of cells cultured on 2D HD-MEAs. By measuring changes in impedance caused by the movement of cardiac organoids, they were able to track the activity of the organoids, so that the microsystem could simultaneously monitor electrophysiological and mechanical movements. This approach is crucial for monitoring the development of cardiac organoids and establishing disease models, as well as for assessing the impacts of drugs on these organoids.

The insertion of 2D microelectrodes directly into organoids allows for deeper and more comprehensive electrophysiological measures. Quadrato et al. [142] used 64-channel high-density silicon microelectrodes to detect isolated spikes from eight-month-old retinal organoids, whereas no spontaneously firing neurons were observed in younger, four-month-old organoids. Similarly, Sharf et al. [148] employed Neuropixels probes, high-density CMOS microelectrode arrays, to explore three-dimensional activity within human brain organoids. By recording extracellular field potentials and action potentials throughout the organoids, they found that theta phase alignment was synchronized with population bursts and localized spiking regions [144].

### 5.2. Advances in 3D MEA

A significant limitation of planar MEAs is their restricted recording area, capturing signals primarily from the outer edges of organoids. To overcome this, researchers have developed 3D HDMEAs that better conform to the complex shapes of organoids:

Buckling and folding strategies for the development of 3D MEAs have been explored. John A. Rogers’ Group: Innovations include 3D silicon electronics fabricated using compressive buckling processes [149] and tensile buckling techniques for 3D architectures [150]. More recently, 3D flexible bioelectronic frameworks were created to match the geometry of neural spheroids, integrating electrical, optical, chemical, and thermal functions at high density [151].

**Figure 2:**
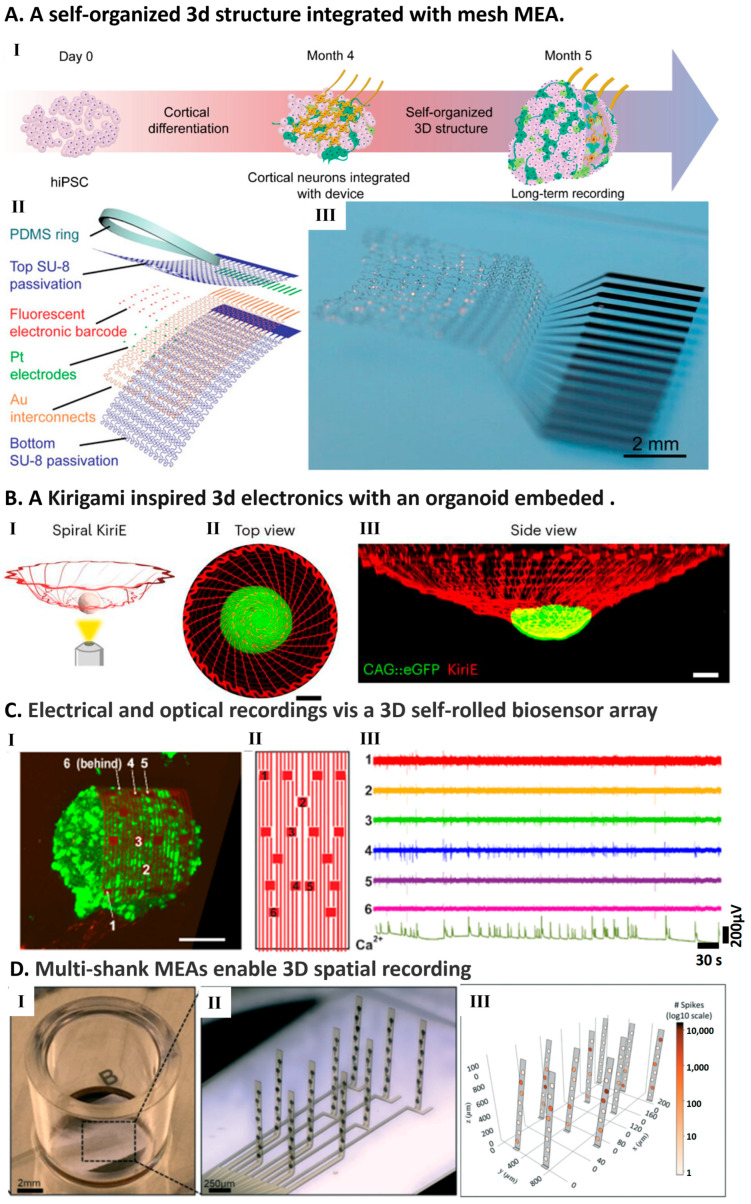
HDMEAs for 3d cultured spheres or organoids. (**A**) “Cyborg” brain organoids integrated with stretchable mesh nanoelectronics [145]. (I) Workflow for co-assembling hiPSCs with the mesh during early development. (II) Device architecture: Pt-black electrodes and gold interconnects are encapsulated within SU-8 layers. The serpentine design confers stretchability, while a PDMS ring defines the cell seeding boundary. (III) Optical view of the released mesh floating in saline. (**B**) Kirigami electronics (KiriE) [29] (I). (II) Top view and (III)side view of confocal imaging demonstrating the intimate embedding of a spiral KiriE probe within a human cortical organoid. Green color shows the CAG::eGFP hCOs and the red lines indicate the spiral KiriE. (**C**) Multimodal monitoring using 3D self-rolled biosensor arrays (3D-SR-BA) [153]. (I) 3D confocal reconstruction of a cortical spheroid (stained with Cal-520 calcium indicator in green) wrapped by the sensor array in red. Scale bar: 100 µm. (II) Layout map of the recording electrodes. (III) Simultaneous traces of spontaneous electrical spikes (voltage) and calcium dynamics (fluorescence intensity) captured from the same tissue region. (**D**) Mechanically actuated 3D vertical electrode arrays [154]. (I) View of a single culture well featuring large ground electrodes. (II) Post-actuation micrograph: hinge regions are plastically deformed to lock the probes in an upright, vertical position. (III) 3D spatial visualization of neuronal spiking activity recorded over a 30 min session. (All figures adapted with permission).

Along similar lines, Cools et al. [152] developed stress-induced self-folding thin-film multielectrode interfaces that wrap around cells, functioning as 3D electrical shells for enhanced interfacing. Kalmykov et al. [153] constructed a 3D self-rolled biosensor array (3D-SR-BA) for cortical spheroids, enabling electrophysiological recordings and calcium imaging (Figure 2C). Recently, Yang et al. [29] introduced kirigami-inspired flexible electronics to enable long-term electrophysiological recording from human neural organoids and assembloids (Figure 2B). The electronics are crafted using SU-8 photoresist with a total thickness of approximately 0.9 μm, featuring microelectrodes with a diameter of 25 microns for precise electrophysiological recordings. The 3D structures are achieved through a kirigami-inspired cutting and folding process, enabling seamless integration with three-dimensional biological assemblies. These flexible systems conform to the complex surfaces of organoids, maintaining stable contact without disrupting growth or function [29].

In addition to buckling or folding strategies, advancements in printing processes and technologies have also expanded the possibilities for the development of 3D MEAs. For example, aerosol jet-printed high-aspect ratio micro-needle electrode arrays have been employed to interface with human cerebral organoids and 3D neurospheroid networks, revealing complex electrophysiological signatures indicative of functional maturation [155,156]. This technique enhances the precision and reliability of data acquisition, offering a window into the bioelectric activities underpinning organoid development.

### 5.3. Vertical and Deep-Implant 3D MEAs

Beyond conformal surface interfacing, another major strategy is to access internal organoid activity more directly through vertical, penetrating, or embedded electrode designs. Soscia et al. [154] fabricated a 256-channel flexible MEA that vertically lifts polyimide probes into 3D configurations using buckling shanks (Figure 2D). This array successfully recorded neural activity from hiPSC-derived neurons and astrocytes embedded in hydrogels over 45 days. Yang et al. [157] introduced neuron-like electronics (NeuE) that mimic neurons’ mechanical properties, supporting organoid development while recording electrophysiological activity. Le Floch et al. [145] developed stretchable mesh nanoelectronics, creating “cyborg organoids” that integrate cell layers into biomaterial scaffolds, supporting organoid growth and enabling long-term single-cell recordings (Figure 2A). Recently, Wu et al. [158] introduced a fully automated robotic system capable of inserting HDMEA into the interior of intact brain organoids, overcoming the depth limitations of traditional planar arrays.

## 6. Advances and Applications of HDMEAs In Vivo Animal Models

HDMEAs have played an important role in extending high-resolution electrophysiology from controlled in vitro systems to physiologically complex in vivo settings. In animal models, these devices have enabled more precise recordings of neural, muscular, and cardiac activity, while also testing key translational factors such as tissue conformity, implantation feasibility, signal stability, and long-term biocompatibility. Recent advances in flexible, stretchable, and multiplexed HDMEAs have further expanded their utility in ECG, ECoG, and EMG.

### 6.1. HDMEAs in ECoG Models

HDMEAs have been widely applied in ECoG to monitor neural activity, both for understanding pathological states and investigating high-level neural functions such as sensory processing and language. Flexible and high-resolution ECoG arrays have been designed to conform to brain surfaces, improving electrode-tissue contact.

Representative studies illustrate this progression. Chiang et al. [19] developed a flexible μECoG array with 294 channels at a density of ~2.6 sites/mm^2^ to study the contribution of the ventrolateral prefrontal cortex (vlPFC) to auditory processing. They also developed a Neural Matrix, a kilo-scale multiplexed electrode array, which features 1008 electrodes across a 9 × 9.24 mm^2^ area, utilizing ultrathin, flexible technology with thermally grown silicon dioxide for robust, long-lasting encapsulation [159]. It integrates powered electronics directly at each electrode to reduce wiring complexity, allowing for high-resolution, stable neural recordings over extended periods, projected to last up to six years, with potential extensions through advanced encapsulation techniques. Kaiju et al. addressed the limitations of low-density arrays by stacking nine 128-channel arrays to achieve 1152 channels at a density of ~11.8 sites/mm^2^, although the integration process involved complex connections to external systems. Paulk et al. [20] utilized a 64-channel PEDOT:PSS electrode with 20-μm diameter electrodes and a 50-μm pitch to detect microscale cortical activity in patients, revealing cortical oscillations and action potentials during craniotomies.

Recent work has also emphasized minimally invasive and functionally precise interfacing. Seo et al. (2023) [21] reported a filamentary multiplexed array with injectable delivery with 256 channels in a 2300 × 300 μm^2^ area. This array features single-neuron-sized electrodes (34 × 7 μm^2^), multiplexed circuits with 1-transistor designs, and rollable contact pads, facilitating minimally invasive delivery via needle injection. In vivo testing demonstrated precise targeting within 100 μm, high signal-to-noise ratios (30.1 ± 10.4 dB), and successful decoding of auditory cortex activity in rats [21]. Furthermore, Uguz and Shepard [70] demonstrated bidirectional communication with L2/L3 pyramidal neurons using HDMEAs for superficial cortical stimulation. Their study showed that electrode size and spacing (down to 15 and 25 μm) confined stimulation laterally, with response depths of 220 to 280 μm. This approach enables precise and localized activation, contrasting with the larger, less specific stimulation fields used in clinical settings. A significant leap in translational technology was demonstrated by Hettick et al. [160], who developed a scalable 1024-channel array that enables minimally invasive implantation without craniotomy, showing promise for human clinical applications.

### 6.2. HDMEAs in EMG Models

HDMEAs have also been applied to EMG, aiding in the diagnosis of muscular disorders such as amyotrophic lateral sclerosis (ALS) and muscular dystrophy (MD), as well as monitoring muscle fatigue. Flexible electrodes improve surface electromyography (sEMG) by enhancing electrode-tissue contact, crucial for detecting weak muscle signals. For example, Choi et al. [22] developed a flexible 8-channel microelectrode array with electrode diameters of 150–300 μm for small-area sEMG recordings. The 150-μm electrodes achieved an RMS voltage of 0.853 mV, providing clear signals for muscle activity analysis. Mao et al. [23] enhanced electrode sensitivity by incorporating amorphous indium–gallium–zinc oxide (a-IGZO) thin-film transistors (TFTs), achieving a 15 dB signal gain with low-voltage operation.

Wearable HDMEAs have expanded the scope of EMG applications. For example, Lu et al. [24] developed a 64-channel HD-sEMG array for personal identification, while Das et al. [161] created a wearable system capable of capturing sEMG signals during epileptic seizures, offering potential diagnostic tools for epilepsy management.

### 6.3. HDMEAs in ECG Models

Cardiovascular diseases, a leading cause of mortality globally, drive the development of HDMEAs for high-resolution cardiac electrophysiology monitoring and therapeutic stimulation. Traditional ECG methods lack spatial resolution, prompting the integration of HDMEAs into cardiac studies.

In zebrafish models, Shih et al. [25] utilized a 3D-carbon nanotube (3D-CNT) microelectrode array with 8 × 8 μm electrodes arranged in a 3 × 3 grid. This array recorded P, QRS, and T-waves with high fidelity, allowing ECG studies at different anatomical regions of the zebrafish heart.

To improve conformal contact with dynamic cardiac tissues, Xiang et al. [26] developed a four-layer stretchable HDMEA with a spatial density of 7.3 sites/mm^2^. This array maintained stable contact during heartbeats, providing high-resolution ECG data from perfused mouse hearts. Lee et al. [27] demonstrated intracellular-like recordings of cardiomyocytes using porous Pt-black electrodes in HDMEAs. Their technique enabled electroporation and intracellular recordings without compromising electrophysiological integrity, facilitating drug response studies with clinically relevant compounds like nifedipine and quinidine.

## 7. Advances in HDMEAs for Human Applications

**Figure 3:**
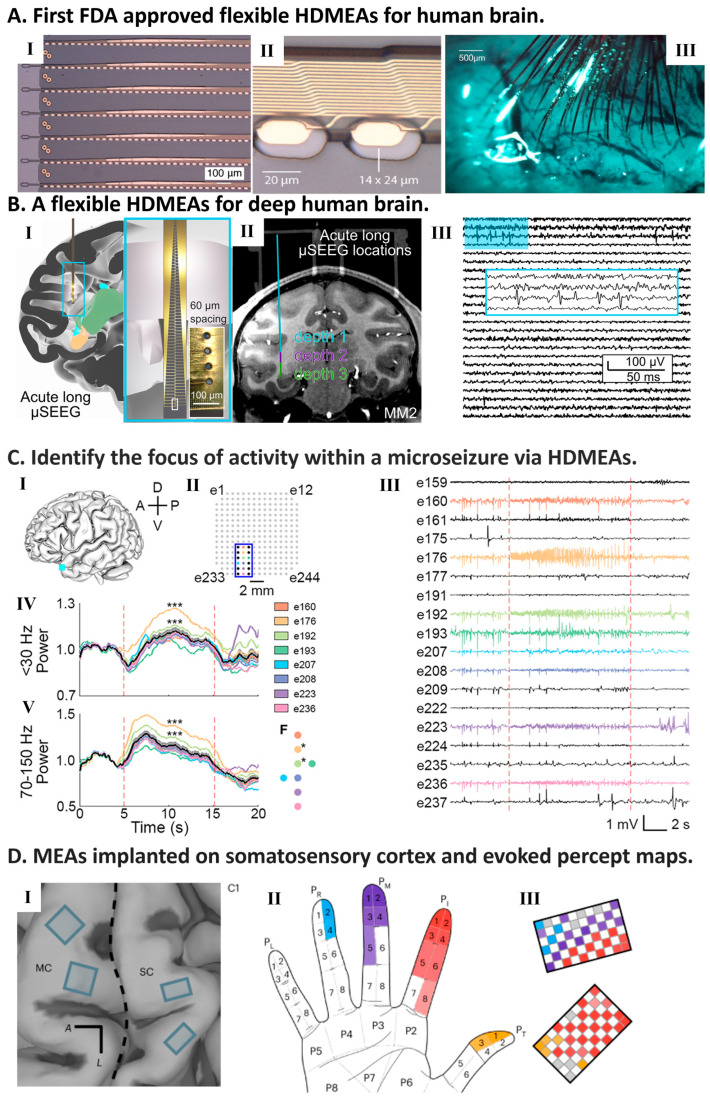
HDMEAs for humans. (**A**) Neuralink’s flexible MEAs, with as many as 3072 electrodes per array distributed across 96 threads [135]. (I) Detail of “Linear Edge” probes featuring 32 contacts with 50 μm spacing. (II) Magnified view of the thread design highlighting the minimal electrode surface area. (III) Intraoperative view showing cortical implantation of threads with reduced vascular disruption. (**B**) Flexible, high-channel-count stereo-electrodes (µSEEG) for deep brain recording [164]. (I) Three-dimensional reconstruction illustrating probe trajectories into parietal and temporal lobes. (II) Coregistered MRI/CT scans confirming electrode depth. (III) Representative single-unit spiking activity (300–6000 Hz filtered) captured from deep tissue. Permission from authors, all rights reserved. (**C**) Spatiotemporal mapping of microseizures using high-density µECoG [163]. (I) Electrode array orientation in patient P4 (A: anterior, P: posterior, D: dorsal, V: ventral). (II) Active channel map on the 244-channel array; colored contacts indicate seizure detection. (III) µECoG voltage traces showing microseizure onset and offset (red dashed lines). (IV,V) Normalized power dynamics in low-frequency (<30 Hz) and high-gamma (70–150 Hz) bands; shaded areas represent SEM (Asterisks (*) signify electrodes which had significantly elevated power compared with the mean. *** *p* < 0.0001). (VI) Spatial cluster of electrodes exhibiting significant power elevation. (**D**) Restoration of tactile sensation via intracortical microstimulation (ICMS) [165]. (I) MRI overlay showing array implantation sites relative to motor (MC) and somatosensory (Area 1) cortices. (II,III) Somatotopic mapping of induced sensations on the hand. Colored zones correspond to specific stimulating electrodes, reflecting the median reported sensations. (All panels adapted with permission).

HDMEAs have extended from experimental systems into clinically relevant settings, particularly in epilepsy diagnostics, intraoperative neuromonitoring, and neuroprosthetic systems. By enabling the recording of neural activity at micro-scale resolution, HDMEAs have facilitated the identification of pathological neural discharges, seizure dynamics, and functional connectivity in perioperative and intraoperative settings. Representative HDMEA platforms used in human and clinical electrophysiology are summarized in Figure 3.

### 7.1. Brain Function Monitoring and Epilepsy Surgery

As shown in Table 2, HDMEA development has also extended into clinically relevant applications, including intraoperative cortical mapping, epilepsy-related electrophysiology, and bidirectional neural interfacing. By enabling microscale recordings of neuronal firing, oscillatory activity, and pathological discharges, these devices can reveal electrophysiological features that are not detectable with conventional clinical electrodes. In earlier years, Schevon et al. [72,162] implanted 96-channel microelectrode arrays (16 mm^2^) into patients with refractory epilepsy and identified microdischarges and high-frequency oscillations within small cortical regions (~200 µm). These findings highlighted microseizures’ significant role in seizure initiation and propagation. Yang et al. [73] employed PEDOT:PSS microelectrode arrays (30 µm electrode diameter, 50 µm pitch) to monitor microscale electrophysiological markers of epilepsy in 30 patients during surgery. Their results demonstrated the ability of HDMEAs to capture microdischarges that could serve as critical markers for epilepsy diagnosis and treatment. Sun et al. [163] further advanced this field by using flexible liquid crystal polymer thin-film µECoG electrode arrays for intraoperative monitoring in nine epilepsy patients (Figure 3C). These high-density µECoG arrays outperformed conventional clinical arrays, detecting microseizures that were otherwise undetectable during both awake and anesthetized states. The broader spatial coverage and enhanced sensitivity of these arrays provide valuable insights into epileptic networks and may improve future epilepsy interventions.

More recently, Keundong Lee and colleagues [164] developed a flexible, scalable, high-channel-count stereoelectrode, which is capable of recording from 128 channels on large glass substrates (18 × 18 cm^2^) using display screen manufacturing techniques (Figure 3B). These electrodes can reach depths of 10 cm within brain tissue, facilitating detailed recordings of local field potentials and single-unit neuronal activities. They have been tested for biocompatibility and shown to cause less tissue damage compared to traditional cylindrical clinical electrodes, enhancing their suitability for long-term implantation and monitoring.

### 7.2. Intraoperative Spinal Monitoring

HDMEAs have demonstrated utility in intraoperative neuromonitoring, offering unparalleled resolution for mapping neural activity in real time during surgical procedures. Russman et al. [56] applied a high-channel-count microelectrode array to the exposed spinal cord during surgery, enabling the creation of two-dimensional maps of responsive channels with submillimeter precision. This approach successfully resolved the electrophysiological midline and recorded both epidural and subdural responses at lower stimulation currents than those typically used in clinical settings. Notably, the sensitivity of HDMEAs allowed the detection of postoperative evoked potential that conventional intraoperative neuromonitoring could not, highlighting their potential for improving surgical outcomes and minimizing neurological risks.

### 7.3. HDMEAs for Neuroprosthetics and Restoration of Function

HDMEAs are also central to emerging neuroprosthetic systems aimed at restoring communication or sensory function. Musk et al. [135] developed a high-bandwidth, scalable brain-machine interface (BMI) featuring an array with 96 flexible polymer threads, each containing 32 electrodes, totaling 3072 electrodes (Figure 3A). These electrodes are crafted from gold and enhanced with conductive polymers and iridium oxide to reduce impedance and boost charge capacity, essential for electrophysiology. The compact device integrates advanced microelectronics for signal amplification and digitization, all contained within a small implantable package measuring less than 23 × 18.5 × 2 mm^3^. This system, notable for its brain tissue compatibility and precision placement via robotic assistance, represents the first flexible HDMEA approved by the FDA for human trials. This platform highlights the translational potential of highly multiplexed, robotically implanted flexible neural interfaces. However, the long-term safety, stability, and clinical effectiveness of such implanted HDMEA systems remain critical questions for future translation. Evaluating the safety and effectiveness of implanted HDMEAs is crucial, with ethical considerations playing a significant role in their application to human subjects.

A clearer demonstration of functional potential is provided by Willett et al. [166], who reported a speech neuroprosthesis using intracortical HDMEAs to decode attempted speech in a participant with ALS. The participant, despite having ALS, achieved a word error rate of 9.1% for a 50-word vocabulary and 23.8% for a 125,000-word vocabulary. Speech was decoded at a rate of 62 words per minute, which is closer to the speed of natural conversation.

In addition to communication restoration, HDMEAs have also supported sensory neuroprosthetic applications. For example, Greenspon et al. [165] demonstrated stable and spatially specific tactile sensations evoked by multi-electrode intracortical microstimulation, highlighting the potential of HDMEA-based systems for sensory restoration (Figure 3D).

## 8. Future Directions

As the integration of HDMEAs in neuroscience and biomedical research continues to evolve, the future of these devices looks promising, driven by rapid advancements in technology and an increasing understanding of biological systems. However, several challenges remain that must be addressed to fully harness the potential of HDMEAs: (1) Material and Design Innovation: Further innovations in biocompatible materials and the microfabrication techniques used to construct HDMEAs are essential. Materials that offer improved durability, flexibility, and physiological compatibility could extend the lifespan and performance of HDMEAs in chronic applications. Additionally, novel designs that enhance signal quality and integration with biological tissues will be critical. (2) Scaling and Integration: Scaling HDMEAs to cover larger areas with higher density without compromising signal quality or causing tissue damage poses significant technical challenges. Advanced computational tools and machine learning algorithms will be critical in managing the vast data generated by these arrays and in developing more sophisticated, real-time analysis techniques. (3) Regulatory and Ethical Considerations: As HDMEAs find more applications in clinical settings, such as in neural prosthetics and personalized medicine, regulatory challenges must be addressed. Ensuring patient safety, data privacy, and ethical considerations in the use of such advanced technologies will be paramount. (4) Commercialization and Accessibility: Moving from research prototypes to commercially available products requires overcoming significant hurdles, including cost reduction, manufacturing scalability, and market acceptance. Partnerships between academia, industry, and regulatory bodies will be crucial in bringing these technologies to the clinic and the broader research community. (5) Interdisciplinary Collaboration: The complexity of HDMEA technology necessitates continued interdisciplinary collaboration. Engineers, neuroscientists, clinicians, and data scientists must work together to design systems that not only advance our understanding of the brain but also lead to practical healthcare solutions.

In conclusion, while HDMEAs offer exciting possibilities for advancing neuroscience and medical diagnostics, their future will depend on concerted efforts in technology development, thoughtful consideration of ethical and regulatory issues, and ongoing collaborative research. As these challenges are addressed, HDMEAs are set to play a pivotal role in the next generation of medical diagnostics and neural interface technologies.

## Figures and Tables

**Table 1:** Classification and characteristics of bioelectric signals detected by MEAs.

Category	Signal Source	Signal Type	Description	Amplitude Range	Frequency Range	Reference
Active Signals from Excitable Cells	Cardiac Pacemaker Cells	ECG	Surface electrical activity reflecting heart rhythm, size, shape, and conduction pathways.	10 µV–4 mV	0.05–150 Hz	Bailey et al. [39]
		Extracellular APs	Local signals from cardiomyocytes or heart surfaces.	100–500 µV	2 Hz–1 kHz	Zhu et al. [40]
	Neurons	APs	High-frequency spikes from neurons, recorded at cellular or axonal levels.	~500 µV	0.1–7 kHz	Robbins et al. [41]
		EEG	Non-invasive scalp recordings of neuron population activity.	5–300 µV	<100 Hz	Alahi et al. [42]
		ECoG	Cortical surface recordings with higher resolution than EEG.	0.01–5 mV	<200 Hz	Buzsaki et al. [43]
		LFP	Extracellular local field potentials reflecting subthreshold activity and synaptic dynamics.	0.01–1 mV	0–200 Hz	Buzsaki et al. [43]
	Neuromuscular Junctions	EMG	Electrical signals from neuromuscular activity; motor unit action potentials (MUAPs).	0.1 µV–5 mV	10–500 Hz	Ng and Reaz [44]; Ali [45]
Cell-Electrode Interaction Signals	Impedance Changes	Dynamic Impedance	Changes in impedance at cell–electrode interface caused by mechanical motion (e.g., contraction).	Variable	50 Hz	Chen et al. [37]
	Other Sources	Adhesion Noise	Fluctuations in impedance or electric fields due to non-electrogenic cell behavior.	Variable	NA	Ell et al. [46]

**Table 2:** Representative state-of-the-art HDMEAs and integrated HDMEA platforms.

References	Electrode Material	Channel Count	Electrode Size	Pitch	Sensing Modality	Stimulation Modality	Applications
[53]	Pt	1024	15 × 15 μm^2^	36 μm	Neural signals	N/A	High-Bandwidth Brain–Computer Interfaces
[21]	Si NM/PI/Au	256	34 × 7 μm^2^	N/A	Electrical	N/A	Neuroscience research and clinical applications
[54]	Pt	26,400	9.3 × 5.4 μm^2^	17.5 μm	Extracellular signals	Electrical	Recording and stimulation of electrogenic cells in vitro
[55]	GC	5 to 8	Ø30–40 µm or 55 × 25 µm oval	100 to 250 µm	FSCV/EIS/CV	N/A	Cultured Neuronal Networks; Acute Brain Slices; Cardiac Cell Cultures
[56]	PtNR	12 × 31	Ø30 μm	350 × 400 µm	SSEPs	N/A	IONM; Functional Midline Mapping; SCI Treatment (Future); Sensory Restoration (Future)
[57]	Au	60	Ø65 µm	265 µm	Extracellular electrophysiology	N/A	Neurospheroids/organoids mapping; human iPSC-derived neural model analysis; Multi-depth electrophysiological recording
[7]	Au/Pt-black	6600	N/A	35 µm	Electrical	N/A	Retinal ganglion cell (RGC)
[58]	Au/PEDOT:PSS/p(C6NDI-T)/p(g2T-TT)	8 × 8/4 × 8	Ø26 μm	40 μm	Electrical	N/A	In vivo LFP recording for large-scale neural interfaces and neuromotor prostheses.
[59]	Activated Iridium Oxide Film (AIROF)	3 × 4	Ø150 µm	350 μm	N/A	Electrical	Retinal Prosthesis, Vision Restoration for AMD and RP
[60]	Pt-black	26,400	9.3 × 5.3 μm^2^	17.5 μm	Whole-array activity scan	N/A	Long-term neuronal tracking; axonal AP propagation; disease modeling
[61]	PtNPs/id-DLG	256	Ø20 μm	350 μm	Electrical	Visual stimulation	Multimodal neural recording and imaging; Ca^2+^ decoding; spatial frequency-band mapping; V1 dynamics
[62]	Pt and graphene	32	Ø10 μm	30 μm	Electrical	Electrical	Retinitis pigmentosa (RP); age-related macular degeneration (AMD); retinal prosthesis
[63]	Au	9	4 × 100 μm	3.7 μm	Redox Cycling	N/A	In vivo dopamine monitoring; microliter-volume chemical analysis
[64]	Pt/Pt-black	26,400	10 × 10 μm	17.5 μm	Extracellular voltage recording	Voltage-controlled stimulation	Input–output studies; neurological disease modeling; bio-inspired AI
[65]	iridium	16	703 μm^2^	100 μm	Extracellular voltage recording	ICMS	Chronic BMI recordings; foreign-body response; cortical depth effects
[66]	EGaIn/PtB	50 × 50 pixels (High-res array); 6 × 6 arrays (In vivo)	Ø20 μm	100 µm (High-res array); 200 µm (In vivo device)	EEPs	Optical	Epiretinal prosthesis; vision restoration in retinal degeneration
[20]	PEDOT:PSS/Cr/Au	32 or 128	Ø20 µm or 30 µm	50 µm, 200 µm, or 800 µm	LFP	N/A	Intraoperative mapping of human cortical surface
[67]	Au, Pt, or Pd,	17	20 × 20/ 30 × 30 μm^2^	5 μm	Neural recording and dopamine (DA) measurements	N/A	High-density neural recording and electrochemical neurotransmitter detection
[68]	Au	256	Ø100 µm or 300 µm	250 µm or 500 µm	Records LFPs, SSEPs, and high-frequency oscillations.	N/A	High-resolution cortical mapping; BCIs; speech prostheses; epilepsy mapping
[69]	Au/Ag/Pt	9	40 × 40 μm^2^	100 μm	Extracellular electrophysiology for recording neural Action Potentials (Spikes)	N/A	In vivo neural recording for neuroscience research and potential neuroengineering therapeutics.
[27]	Pt-black	26,400	5.5 × 9.3 μm^2^ Ø4 μm	17.5 μm	Extracellular FP recording; electroporation-mediated intracellular-like AP recording	Electrical/Electroporation	Intracellular-like recording from hiPSC-CMs; cardiotoxicity screening; cardiac electrophysiology; tissue slices and 3D organoids
[70]	PEDOT:PSS/Au	60	Ø15 µm	40 μm	Electrophysiology	Electrical	High-resolution cortical stimulation; bidirectional BMIs; neural circuit studies
[71]	CMOS	900	9.3 μm × 5.45 μm	17.5 μm	Extracellular spike recording; extracellular footprint extraction	N/A	Tracking axon initial segment plasticity
[38]	Au	512 sensing and 4 pacing electrodes	Ø100 μm	420 µm	FPM EIS	Integrated Multipoint Electrical Stimulation	Cardiac electrophysiology and contraction monitoring; rotor analysis; cardioactive drug testing
[26]	Cr/Au	32	N/A	200 µm	Electrophysiological	N/A	Spatiotemporal mapping of electrocardiogram
[72]	Pt-coated silicon	96	Ø35 to 75 μm	400 µm	Multichannel extracellular recording of LFPs, HFOs, and MUA	N/A	High-resolution mapping of epileptic brain activity to identify seizure-onset zones for surgical guidance
[73]	PEDOT:PSS	128	Ø30 µm	50 μm	LFP	N/A	Mapping the microscale spatial dynamics of epileptiform activity in the human brain.
[31]	PEDOT:PSS/Pt/Au/Pt	16 + 2	Recoding microelectrodes 33 μm × 33 μm/chemical electrodes 40 μm × 400 μm	50 μm	electrical/chemical	Electrical	Neural circuitry dissection; neural network connectivity

Glassy carbon (GC); Eutectic gallium–indium alloy (EGaIn); Gold (Au), platinum (Pt); palladium (Pd); Somatosensory Evoked Potentials (SSEPs), intracortical microstimulation (ICMS), Field Potential Monitoring (FPM), Electrochemical Impedance Spectroscopy (EIS), Extracellular recording of local Field Potentials (LFP), High-Frequency Oscillations (HFO); Multi-Unit Activity (MUA); Extracellular recording of electrically evoked potentials (EEPs).

## Data Availability

No new data were created or analyzed in this study. Data sharing is not applicable to this article.
